# Plant evolution can mediate negative effects from honey bees on wild pollinators

**DOI:** 10.1002/ece3.6207

**Published:** 2020-04-12

**Authors:** James R. D. Milner, Elias H. Bloom, David W. Crowder, Tobin D. Northfield

**Affiliations:** ^1^ Centre for Tropical Environmental and Sustainability Studies College of Science and Engineering James Cook University Cairns Qld Australia; ^2^ Department of Entomology Michigan State University East Lansing MI USA; ^3^ Department of Entomology Washington State University Pullman WA USA; ^4^ Department of Entomology Tree Fruit Research and Extension Center Washington State University Wenatchee WA USA

**Keywords:** biodiversity, honey bees, mathematical model, mutualism, plant evolution, wild pollinators

## Abstract

1. Pollinators are introduced to agroecosystems to provide pollination services. Introductions of managed pollinators often promote ecosystem services, but it remains largely unknown whether they also affect evolutionary mutualisms between wild pollinators and plants.
2. Here, we developed a model to assess effects of managed honey bees on mutualisms between plants and wild pollinators. Our model tracked how interactions among wild pollinators and honey bees affected pollinator and plant populations.
3. We show that when managed honey bees have a competitive advantage over wild pollinators, or a greater carrying capacity, the honey bees displace the wild pollinator. This leads to reduced plant density because plants benefit less by visits from honey bees than wild pollinators that coevolved with the plants.
4. As wild pollinators are displaced, plants evolve by increasing investment in traits that are attractive for honey bees but not wild pollinators. This evolutionary switch promotes wild pollinator displacement. However, higher mutualism investment costs by the plant to the honey bee can promote pollinator coexistence.
5. Our results show plant evolution can promote displacement of wild pollinators by managed honey bees, while limited plant evolution may lead to pollinator coexistence. More broadly, effects of honey bees on wild pollinators in agroecosystems, and effects on ecosystem services, may depend on the capacity of plant populations to evolve.

## Graphical abstract

Introduced pollinators can displace wild pollinators from wild plant habitats. Our results show plant evolution can promote displacement of wild by introduced pollinators, while limited plant evolution may lead to pollinator co‐existence. More broadly, effects of introduced on wild pollinators in agroecosystems, and effects on ecosystem services may depend on the capacity of plant populations to evolve.

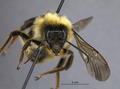

## 1 INTRODUCTION

Pollinators provide billions of dollars in global pollination services annually (Goulson, Nicholls, Botias, & Rotheray, 2015; Losey & Vaughan, 2006) and promote plant biodiversity (Burkle, Marlin, & Knight, 2013). The European honey bee, *Apis mellifera mellifera*, is widely considered the most agriculturally important pollinator (Rucker, Thurman, & Burgett, 2012); honey bees provide pollination services in agroecosystems that are often additive to those provided by wild insects, at least over relatively short time scales (Garibaldi et al., 2013). While pollinators can exist as mutualistic partners, alongside pesticides and pathogens (Goulson et al., 2015), honey bees may reduce wild pollinator diversity and threaten coevolved plant–pollinator mutualisms through indirect interactions (Magrach, Gonzalez‐Varo, Boiffier, Vila, & Bartomeus, 2017; Paini & Roberts, 2005; Sugden, Thorp, & Buchmann, 1996; Thomson, 2006). There is, thus, a need to assess potential effects of honey bees on populations of wild pollinators (Geldmann & Gonzalez‐Varo, 2018).

Negative effects of honey bees on wild pollinators may stem from aggressive interactions at floral resources (Cairns, Villanueva‐Gutierrez, Koptur, & Bray, 2005; Geslin et al., 2017), resource competition (Geldmann & Gonzalez‐Varo, 2018; Steffan‐Dewenter & Tscharntke, 2000; Torne‐Noguera, Rodrigo, Osorio, & Bosch, 2016), and through displacement of wild species to low‐reward nutrients (Magrach et al., 2017; Schaffer et al., 1983; Thorp, 1996). While much of this research has been focused on interactions with other bees, their impacts may be widespread, including negative effects on Dipteran (true fly) pollinators and other flying insects (Lindstrom, Herbertsson, Rundlof, Bommarco, & Smith, 2016). However, individual honey bees are often less effective pollinators (Garibaldi et al., 2013) and contribute less to stability of pollination services than wild insects (Garibaldi et al., 2011). This lower impact may be due to honey bees generally exhibiting low floral fidelity, which reduces conspecific pollen deposition (Geslin et al., 2017). Thus, while honey bees may often displace wild pollinators because they are managed to reach high densities, the displacement of wild pollinators may often come at a cost to pollination services and plant fecundity.

Given that honey bees can negatively affect wild pollinators and plants (Geldmann & Gonzalez‐Varo, 2018; Geslin et al., 2017; Mallinger, Gaines‐Day, & Gratton, 2017), there is a need to assess the evolutionary implications and mechanisms for how managed honey bees integrate into natural systems, including the potential for plants to adapt. Recent research found that plants near apiaries evolved to produce less nectar than those farther away, presumably due to reduced need to reward pollinators when honey bees are highly abundant (Mu et al., 2014). The plants nearer to apiaries instead invest more heavily in flowers than plants far from apiaries, suggesting that honey bees induced a change in strategies for pollination investment across the plant population (Mu, Wu, Yang, Huang, & Grozinger, 2018). While these studies focused on general traits (e.g., nectar quantity, rather than composition), other research suggests that volatile differences on closely related plants with similar flower morphology can have dramatic impacts on the species of pollinators attracted to the plants (Gong et al., 2015). In selection experiments, Sahli and Conner (2011) observed that variation in pollinator exposure can drive variations in rapid evolution, with honey bees stimulating greater anther exsertion than smaller wild bees, and reduced stamen dimorphism relative to bumblebees. Together, these studies suggest that managed honey bees may induce evolutionary changes in plants that impact wild pollinators.

Pollination theory suggests plants should evolve to attract the most abundant pollinator (Sargent & Otto, 2006), and plants have indeed been shown to exhibit rapid evolutionary adaptations to attract particular pollinator species by altering floral traits such as UV reflectance and floral volatiles (Gervasi & Schiestl, 2017). Such evolutionary responses to pollinators may have dramatic effects on the abundance of both plants and pollinators. For example, the evolution of a mutualist partner can promote species invasions by investing more in the mutualism with the newly abundant invader (Jones & Gomulkiewicz, 2012). Evolution of a mutualist partner can also exaggerate environment increases in the abundance of a species (Northfield & Ives, 2013). However, when the evolutionary benefit to one species comes at a cost to a mutualist partner (e.g., pollinators evolving a better ability to deplete a flower of its resources), evolution may be expected to alleviate that cost by reducing the environmental effects on the mutualist partner's abundance (Northfield & Ives, 2013). While these studies focused on pair‐wise evolution between mutualistic partners, theoretical research on complex pollination networks suggests that indirect interactions can play an important role in altering the evolution of species embedded within a network (Guimaraes, Pires, Jordano, Bascompte, & Thompson, 2017). Thus, to better understand the long‐term implications of honey bees on existing plant–pollinator interactions, general theory is needed that focuses on all three species: the wild plant, the wild pollinator, and the honey bee.

Here, we develop a model to assess ecological and evolutionary implications of introducing a generalist pollinator like the honey bee on an existing wild pollinator–plant mutualism. We considered three ways honey bees could influence wild pollinator abundance and assessed the potential for plant–pollinator trait evolution to mitigate these effects. First, honey bees may impact wild pollinators through per capita competitive effects that are stronger than reciprocal competitive effects from the wild pollinator, such as honey bees deterring wild pollinators from floral resources (Gross et al., 2019; Paini, 2004). Second, honey bees can reach higher abundance despite similar per capita effects of competition, potentially from supplemental resources from beekeepers. Honey bees then have a gross competitive advantage, potentially providing a more widespread pollination service (Hudewenz & Klein, 2015; Thomson, 2004; Torne‐Noguera et al., 2016). Finally, we consider that managed honey bees may benefit more from a flower visit than the wild pollinator, which may make the honey bees more efficient feeders. This could occur if honey bees are more efficient at using nutrients from the flower or has complementary nutrients available such that only the nutrients provided by the flower are limiting. While at least the first two of these mechanisms can be determined through fitting competition models to experimental data (Inouye, 2001), distinguishing between these mechanisms has not been considered experimentally (Mallinger et al., 2017). Thus, we present each mechanism separately, along with resulting evolutionary changes, so hypotheses can be tested for each and scenarios identified which allow for the coexistence of managed honey bees and wild pollinators.

## 2 MATERIALS AND METHODS

### 2.1 Model description

Although plant–pollinator networks are often speciose and complex (Geslin et al., 2017), we focus on a simplified network to identify mechanisms that may occur in larger networks. We assumed each species is a facultative mutualist, where plants may be self‐pollinated, and pollinators have alternative resources besides the focal plant; such a scenario will likely occur as a single module nested within a more complex ecosystem.

Our model assumed pollinators compete for floral resources through behavioral interference as a function of abundance (e.g., Mallinger et al., 2017). We assume pollinator visitation rates are determined by investment in traits by plants and pollinators. For plants, this includes showy flower petals, nectar, volatiles, or other traits (Rosenheim, Williams, & Schreiber, 2014). For pollinators, this may be a propensity to locate focal plants, such as sensitivity to floral volatiles or colors. We characterized the range of traits important for pollination as one representative trait for each species.

Our model approximated a geospatially mixed environment with inter and intraspecific interactions between panmictic populations. When considering evolution, we assumed there is limited phenotypic variance arising from environmental factors (Abrams, 2001). We acknowledge that this assumption does not hold for strong selection, long temporal periods without mutations, and small populations with high genetic drift (Abrams, 2001). Finally, we assumed discrete time potentially arising from the effects of distinct circadian rhythms on pollinator behavior (Bloch, Bar‐Shai, Cytter, & Green, 2017) and logistic growth of each species in the absence of the mutualism to account for intraspecific competition.

### 2.2 Ecological model

We developed a three species, discrete‐time mutualism model with a plant and two pollinators. We assumed plant reproduction increases with pollinator visits, and pollinators use resources provided by plants in ways that promote pollinator reproduction. While pollinator reproduction can increase directly from plant visits, like when flowers are visited for oviposition (Pellmyr & Huth, 1994), we focused on the more common scenario where plants provide resources like nectar and pollen that benefit pollinators. However, modelling pollinator reproduction as directly proportional to plant visits led to the same conclusions described below (data not shown).

The abundance of the plant at time *t* + 1 was,

(1) $$P_{t+1}=P_{t}e^{r_{P}-\frac{r_{P}}{b_{P}}P_{t}+\sum_{i=1}^{2}\theta_{Pi}a_{i}N_{i,t}}$$

where *N*_i,_ *_t_* represents wild pollinator (*i* = 1) or managed honey bee (*i* = 2) abundance, respectively, and *r*_P_ is the plant intrinsic growth rate. The per capita plant visitation rate for pollinator species *i* is a function of the traits of both species, *a_i_* = ln(*v_i,t_* + *d_i_u_i,t_* + 1)/(*a*_0_ *_i_* + *P_t_*), which is a decreasing function of *P_t_*, where *v_i,t_* represents the trait of pollinator species *i* and *u_i,t_* represents the plant trait governing investment in the mutualism with pollinator species *i*. The parameter *d_i_* scales the plant traits relative to pollinator traits for pollinator species *i*. The likelihood of an individual plant being visited thus decreases as plant abundance increases. This negative density dependence arises from limits to the potential number of flowers visited by each pollinator individual; *a*_0_ *_i_* influences this limitation (Real, 1977).

For pollinators, traits (*v_i_*) represent characteristics associated with locating the focal plant as opposed to other plant species. Similarly, plant traits represent characteristics that attract a particular pollinator. The parameter *θ*_P_ *_i_* describes the benefit to the plant of each visit by pollinator species *i*. The plant carrying capacity, *b*_P_, decreases linearly with each trait, *k*_P_ *– f*_P1_ *u*_1_ *_,t_ – f*_P2_ *u*_2_ *_,t_*, such that investing in the trait comes at a cost for density‐dependent growth (Northfield & Ives, 2013). The single species carrying capacity is *k*_P_ when trait values are zero and decreases with plant trait *i* at rate *f*_P_ *_i_*. These costs associated with plant traits avoid unbounded growth and runaway selection (May, 1982; Northfield & Ives, 2013). We incorporated the cost of investing in pollinator attraction in the density‐dependent growth parameter, since applying this cost to the intrinsic growth rate only slows the speed of dynamics, but still leads to unbounded growth and runaway selection in our initial analyses.

Pollinator reproduction increased with consumption of pollination rewards (e.g., nectar, pollen), which the plant produces at a constant rate, *r_X_*. The abundance of pollination rewards at time *t* + 1, *X_t_*_+1_, is then equal to the sum of production by plants at time *t*, and the proportion not consumed by pollinators at time *t*,

(2) $$X_{t+1}=r_{X}P_{t}+X_{t}e^{-\sum_{i=1}^{2}a_{i}N_{i,t}}$$

The abundance of wild pollinators (*i* = 1) and managed honey bees (*i* = 2), at time, *t* + 1, is,

(3) $$N_{1,t+1}=N_{1,t}e^{r_{1}-\frac{r_{1}}{b_{1}}\left(N_{1,t}+c_{1}N_{2,t}\right)+\left(\theta_{1}a_{1}X_{t}\right)}$$

(4) $$N_{2,t+1}=N_{2,t}e^{r_{2}-\frac{r_{2}}{b_{2}}\left(N_{2,t}+c_{2}N_{1,t}\right)+\left(\theta_{2}a_{2}X_{t}\right)}$$

where *θ_i_* represents benefits received by pollinator species *i* from consuming pollination rewards during each plant visit. The per capita effect of competition on pollinator *i* is *c_i_*, and *r_i_* is the intrinsic pollinator growth rate in isolation. As with *b*_P_, we assumed that the carrying capacity for pollinator *i*, *b_i_*, decreases linearly with its trait, such that *b_i_* = *k_i _– f_i_v_i,t_*. We focused on a scenario where plants benefited less from each honey bee visit than each wild pollinator visit (*θ*_P1_ *> θ*_P2_), as may occur when honey bees, which are broad generalists, transfer less pollen to flowers (Garibaldi et al., 2013). Relaxing this assumption would benefit the plant after pollinator displacement, and likely indirectly benefit the managed honey bee from higher plant abundance.

### 2.3 Evolutionary change

To model evolution, we followed a simplified quantitative genetics approach, where trait change equals the derivative of fitness with respect to the trait, divided by mean fitness, multiplied by the genetic variance for the trait (Abrams, 2001; Abrams & Matsuda, 1997). For plants, genetic variances are $\sigma_{P1}^{2}$ and $\sigma_{P2}^{2}$ , for traits 1 and 2. We assume these genetic variances are independent. Thus, the evolving plant trait values produced for the relationship with the wild pollinator, *u*_1_ *_,t_*, and the managed honey bee, *u*_2_ *_,t_*, are:

(5) $$u_{1,t+1}=u_{1,t}+\frac{1}{W_{P}}\frac{\delta W_{P}}{\delta u_{1}}\sigma_{P1}^{2}$$

(6) $$u_{2,t+1}=u_{2,t}+\frac{1}{W_{P}}\frac{\delta W_{P}}{\delta u_{2}}\sigma_{P2}^{2}$$

and *W_P_* represents mean plant fitness.

The trait values of the wild pollinator, *v*_1,_ *_t_*, and managed honey bee, *v*_2,_ *_t_*, are then:

(7) $$v_{1,t+1}=v_{1,t}+\frac{1}{W_{N1}}\frac{\delta W_{N1}}{\delta v_{1}}\sigma_{1}^{2}$$

(8) $$v_{2,t+1}=v_{2,t}+\frac{1}{W_{N2}}\frac{\delta W_{N2}}{\delta v_{2}}\sigma_{2}^{2}$$

where *σ_i_* ^2^ represents the genetic variances for pollinator *i*, and *W_N_*_1_ and *W_N_*_2_ represent the mean fitness for pollinator 1 and 2, respectively. The evolving plant trait values, *u*_1_ *_,t_* and *u*_2_ *_,t_*, in the plant species are given by

(9) $$u_{1,t+1}=u_{1,t}+\frac{1}{W_{P}}\frac{\delta W_{P}}{\delta u_{1}}\sigma_{P1}^{2}=u_{1,t}+\left[\left(-\frac{r_{P}P_{t}f_{P1}}{b_{P}{\left(u_{1,t},u_{2,t}\right)}^{2}}\right)+\frac{\theta_{P1}N_{1,t}d_{1}}{\left(v_{1,t}+d_{1}u_{1,t}+1\right)\left(a_{01}+P_{t}\right)}\right]\sigma_{P1}^{2}$$

(10) $$u_{2,t+1}=u_{2,t}+\frac{1}{W_{P}}\frac{\delta W_{P}}{\delta u_{2}}\sigma_{P2}^{2}=u_{2,t}+\left[\left(-\frac{r_{P}P_{t}f_{P3}}{b_{P}{\left(u_{1,t},u_{2,t}\right)}^{2}}\right)+\frac{\theta_{P2}N_{2,t}d_{2}}{\left(v_{2,t}+d_{2}u_{2,t}+1\right)\left(a_{02}+P_{t}\right)}\right]\sigma_{P2}^{2},$$

representing the trait produced for the relationship with the wild pollinators and managed honey bees. Thus, we model the rate of evolution to be proportional to *r*_P_/[*b*_P_(*u*_1,_ *_t_*,*u*_2,_ *_t_*)]^2^, the ratio of intrinsic growth rate to carrying capacity squared.

The trait value of the wild pollinator, *v*_1_, and managed honey bee, *v*_2_, are given by

(11) $$v_{1,t+1}=v_{1,t}+\frac{1}{W_{N1}}\frac{\delta W_{N1}}{\delta v_{1}}\sigma_{1}^{2}=v_{1,t}+\left[\left(-\frac{r_{1}\left(N_{1,t}+c_{1}N_{2,t}\right)f_{1}}{b_{1}{\left(v_{1,t}\right)}^{2}}\right)+\frac{\theta_{1}X_{t}}{\left(v_{1,t}+d_{1}u_{1,t}+1\right)\left(a_{01}+P_{t}\right)}\right]\sigma_{1}^{2}$$

(12) $$v_{2,t+1}=v_{2,t}+\frac{1}{W_{N2}}\frac{\delta W_{N2}}{\delta v_{2}}\sigma_{2}^{2}=v_{2,t}+\left[\left(-\frac{r_{2}\left(N_{2,t}+c_{2}N_{1,t}\right)f_{2}}{b_{2}{\left(v_{2,t}\right)}^{2}}\right)+\frac{\theta_{2}X_{t}}{\left(v_{2,t}+d_{2}u_{2,t}+1\right)\left(a_{02}+P_{t}\right)}\right]\sigma_{2}^{2}$$

where *σ_i_* ^2^ represents the genetic variances for pollinator *i*, and the carrying capacity *b*_1_(*v*_1_, t) and *b*_2_(*v*_2_, t) is modelled as *k*_1_ – *f*_1_ *v*_1_ and *k*_2_ – *f*_2_ *v*_2_ for wild pollinators and managed honey bees, respectively, as described in the ecological model (see Appendix S1 for derivation). The parameters *a*_0_ *_i_* represent the half‐saturation constant for pollinator *i*, describing the plant density at which half of the maximum number of plants are visited.

### 2.4 Model analysis

We used simulations to assess how a managed honey bee may affect a coevolved plant–pollinator mutualism. We started at the two‐species eco‐evolutionary equilibrium (i.e., the plant and wild pollinator), then introduced the honey bee at the same abundance as the wild pollinator and tracked the trajectories until a new equilibrium was reached. First, to demonstrate the role of plant evolution, we consider two scenarios: (a) where plants continue to evolve after the introduction of the second pollinator and (b) plant traits are considered fixed when the honey bee is introduced, such that the plant traits are held at the two‐species (plant and wild pollinator) eco‐evolutionary equilibrium. Varying the initial density of the managed honey bee had little effect on the final equilibrium, but introducing the pollinator at a high abundance reflected scenarios where beekeepers introduce honey bees into new environments in high numbers. We assumed initial trait values related to the wild pollinator–plant mutualism (*u*_1_, *v*_1_) were double the managed honey bee–plant mutualism (*u*_2_, *v*_2_) trait values. While empirical data are generally not available to directly parameterize the model, we used parameter values that allowed realistic abundances of each species and allowed coexistence of each species. For example, particularly high trait costs would not have allowed mutualism, and particularly strong differences in pollinator performance would not have allowed coexistence, so we do not present simulations with these parameter values.

We assessed effects of three parameters on the final trait values and densities of the pollinators and plant species. Specifically, we simulated the model for different values of *c*_1_ (0.25*c*_2_ to 2*c*_2_) to vary direct competitive effects on the wild pollinator from the honey bee*, k*_2_ (0.5 *k*_1_ to 2.0 *k*_1_) to vary the honey bee carrying capacity, and *θ*_2_ (0.5*θ*_1_ to 4*θ*_1_) to vary the per‐visit benefit to the honey bee. Each of these parameters was considered because it might mediate displacement of wild pollinators by managed bees. Model parameters were selected to allow the system to reach equilibrium and avoid positive feedback loops that lead to unbounded growth, through sufficient density‐dependent intra and interspecific competition (May, 1982).

We also evaluated situations where the managed honey bee had a competitive advantage per capita (*c*_1_ > *c*_2_), or a higher carrying capacity (*k*_2_ > *k*_1_). These situations are inspired by two potential types of phenomena that could influence direct competition from honey bees on wild pollinators: strong per capita competition from honey bees, and numerical advantages of honey bees driven by the resources provided by beekeepers. We incrementally increased the direct cost to the honey bee (*f*_2_) or plant (*f_P_*_2_) of mutualism investment. All simulations ran for 200,000 time steps to reach equilibrium.

## 3 RESULTS

### 3.1 Effects of evolution on displacement

**FIGURE 1:**
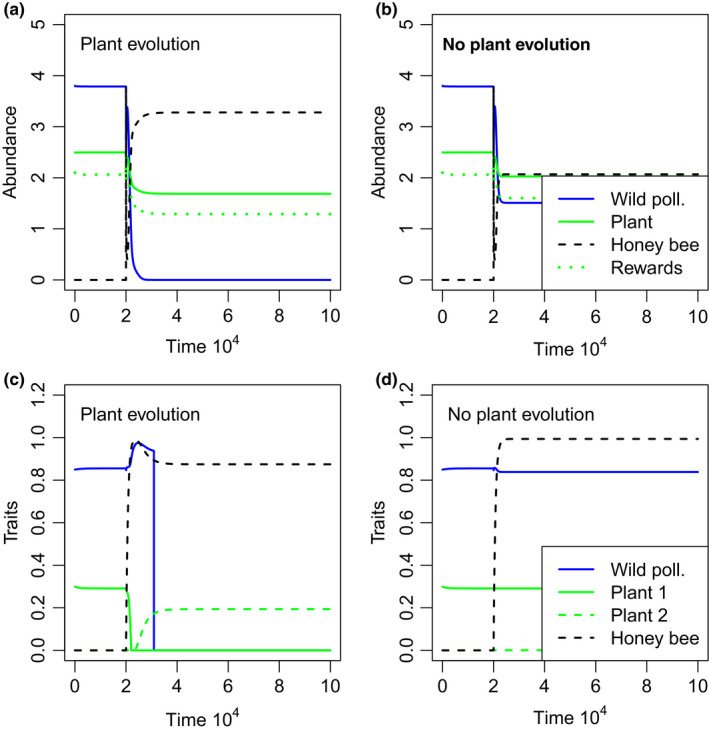
Time series of abundance (a, b) and trait values (c, d) for the wild pollinator (blue lines), plant (green solid lines), the managed honey bee (black dashed lines) and pollination rewards (panels a and b only), *X_t_* (green dotted lines). Panels c and d show the two plant traits associated with each pollinator (matched by line type). Simulations were run for 200,000 time steps with only the plant and wild pollinator and initial traits near evolutionary equilibrium, after which the managed honey bee was introduced and the model was simulated for another 800,000 time steps. In panels a and c, all species continue to evolve after the introduction of the pollinator. In panels b and d, we assume fixed plant traits (i.e., no plant evolution) after the introduction of the honey bee. Legends in panels b and d apply to a and c also. The honey bee is introduced at the same density as the wild pollinator, and the initial honey bee trait value and value of the plant trait for attracting honey bees were each set to 0.001 (i.e., *v*_2,_ *_t_* = *u*_2,_ *_t_* = 0.001 for *t* = 20,000). The competition coefficients for effects on the wild (*c*1) and managed pollinators (*c*2) were set at 1 and 0.75, respectively. All other parameters were as described in Table 1

In our simulations where managed honey bees outcompete the wild pollinator, we found that evolution of the plant exaggerates the decline of the wild pollinator (Figure 1a,b). Indeed, in some scenarios where competitive interactions are weak enough to allow coexistence of the two species, plant evolution could mediate the displacement of the wild pollinator (Figure 1). This increased wild pollinator extinction risk is due to a change in plant investment, from investing in attracting wild pollinators to investment in the honey bee, stimulated by higher densities of the honey bee than wild pollinator (Figure 1). Initially, when managed honey bees induce less plant investment in wild pollinator attraction, the wild pollinator invests more in the mutualism, partially making up for the reduced investment by the plant (Figure 1c). However, eventually, the wild pollinator investment also declines before it goes extinct (Figure 1a,c). In the absence of plant evolution, there is little evolutionary change in either pollinator species (Figure 1b,d), suggesting that evolutionary dynamics largely affected the plant, rather than pollinator evolution (Table 1).

**TABLE 1:** Model variables and parameters and standard values used in simulations. Some parameters were varied from the values below as part of the analysis, and such variation is described in the associated figures

Symbol	Description	Value
*N*_1,_ *_t_*	Wild pollinator abundance at time *t*	Variable
*N*_2,_ *_t_*	Managed honey bee abundance at time *t*	Variable
*P_t_*	Plant species abundance at time *t*	Variable
*v*_1,t_	Wild pollinator trait to attract plant at time *t*	Variable
*v*_2,t_	Managed honey bee trait to attract plant at time *t*	Variable
*u*_1,t_	Plant trait to attract wild pollinator at time *t*	Variable
*u*_2,t_	Plant trait to attract managed honey bee at time *t*	Variable
*r*_1_	Intrinsic growth rate of wild pollinator	0.01
*r*_2_	Intrinsic growth rate of managed honey bee	0.01
*r_p_*	Intrinsic growth rate of plant	0.01
*r_X_*	Pollen production rate	0.5
*c*_1_	Direct competitive effects on the wild pollinator species	0.75
*c*_2_	Direct competitive effects on the managed honey bee species	0.75
*f*_1_	Cost of wild pollinator species investing in the plant	0.5
*f*_2_	Cost of managed honey bee species investing in the plant	0.5
*f*_P1_	Cost of plant investing in the wild pollinator species	0.2
*f*_P2_	Cost of plant investing in the managed honey bee species	0.2
*σ*_1_	Genetic variance of wild pollinator	0.05
*σ*_2_	Genetic variance of managed honey bee	0.05
*σ*_P_ *_i_*	Genetic variance of plant trait *i*	0.05
*k*_1_	Carrying capacity of wild pollinator	1.5
*k*_2_	Carrying capacity of managed honey bee	1.5
*k_P_*	Carrying capacity of plant	0.5
*d*_1_	Effects of plant traits on wild pollinator visits relative to wild pollinator traits	0.8
*d*_2_	Relative effects of plant traits on managed honey bee visits	0.8
*θ*_1_	Wild pollinator benefit from plant	0.05
*θ*_2_	Managed honey bee benefit from plant	0.05
*θ*_P1_	Plant benefit from wild pollinator	0.5
*θ*_P2_	Plant benefit from managed honey bee	0.25
*a*_01_	Constant reducing wild pollinator visitation	0.5
*a*_02_	Constant reducing managed honey bee visitation	0.5

**FIGURE 2:**
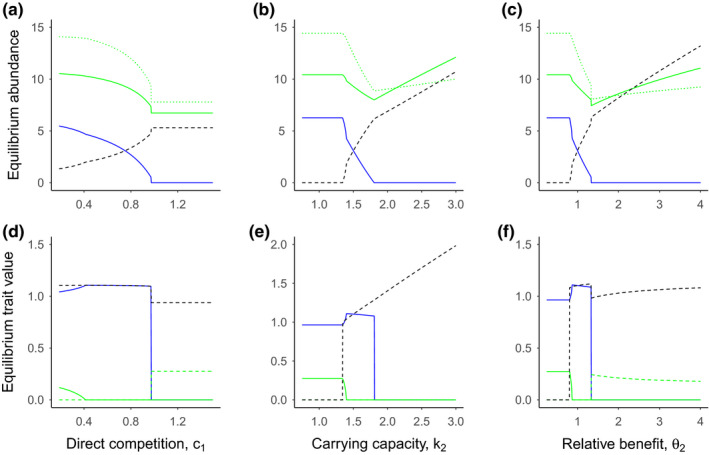
Effects of competition, carrying capacity, and benefits on pollinator and plant abundance and traits. Patterns of equilibrium abundance (a, b, c), and equilibrium trait values (d, e, f) for the wild pollinator (blue lines), plant (green solid lines), the managed honey be (black dashed lines), and pollination rewards (panels a, b, and c only), *X_t_* (green dotted lines). Panels d, e, and f show the two plant traits associated with each pollinator (matched by line type). Panels a and d represent final equilibrium abundances (a) and traits (d) for a range of direct competition effects on the wild pollinator from the honey bee (*c*_1_), where all three species are introduced with abundance 0.05, and trait value 0.005. The honey bee was introduced after 2,000 time steps, and then tracked for a total of 200,000 time steps to ensure equilibrium had been reached. Panels b and e represent final equilibrium abundances (b) and traits (e) after the introduction of the pollinator for a range of carrying capacities for the managed honey bee (*k*_2_). Panels c and f represent final equilibrium abundances (c) and traits (f) for a range of mutualism benefits received by the managed honey bee (*θ*_2_). Other parameter values are presented in Table 1

To evaluate the effects of ecological interactions and variation in their intensity, we considered the impacts of various parameters on the final equilibrium abundance and trait values of the pollinators and plant. In these evaluations, we found that coexistence of both pollinators occurred when per capita effects of competition on the wild pollinator were less than 1.0 (*c*_1_ < 1.0) for one parameter set, such that per capita competition from the managed honey bee was weaker than intraspecific competition on the wild pollinator (Figure 2a). Increases in competition from honey bees were also associated with decreases and increases in plant traits associated with wild pollinators and honey bees, respectively. In addition to competitive interactions, we also considered two scenarios where managed honey bee abundance can increase, by either increasing their carrying capacity (*k*_2_) or the benefit received by the honey bee from the plant (*θ*_2_). Each of these gives the managed honey bee a numerical competitive advantage over the wild pollinator (Figure 2). In these two scenarios, where either the honey bee carrying capacity (*k*_2_), or the benefit received by the honey bee from the plant (*θ*_2_) relative to that received by the wild pollinator (*θ*_1_) was increased, a similar pattern in species abundances and trait values emerged (Figure 2). For low values, wild pollinator remained the dominant mutualistic partner, with an intermediate parameter range with coexistence and the managed honey bee displaced the wild pollinator at higher values (Figure 2a–c). In each case, plant traits broadly reflected the relative abundances of the two pollinators, with plant traits declining or increasing with the abundance of the associated pollinator (Figure 2).

The changes in final plant traits can be explained by the impacts of pollinator abundance on selection pressure for each plant trait. As the abundance of the wild pollinators decreases, selection pressure for attracting wild pollinators also decreases, driving evolution of reduced plant traits associated with wild pollinators, and increased trait values associated with managed honey bees (Figures 1 and 2). Interestingly, for sufficiently large honey bee carrying capacity (*k*_2_), we observed high trait values of the honey bees, along with very high honey bee abundance (Figure 2b,e). In this case, plant traits were zero, presumably because high pollinator abundance meant no need for investment in pollinator attraction (Figure 2b,e). When this carrying capacity was high enough, we observed unbounded growth of the plant and managed honey bee (data not shown). Plant investment was also relatively low when pollinators coexist at similar abundances, and pollinator abundance and investment by each species are high (e.g., *c*_1_ = 0.9 in Figure 2a,c).

**FIGURE 3:**
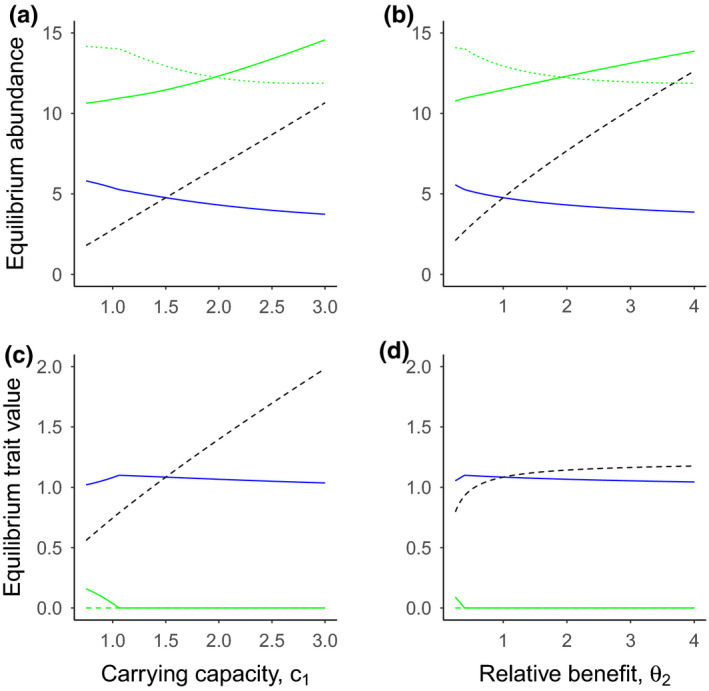
Effects of carrying capacity and benefits on pollinator and plant abundance and traits when pollinators do not compete directly. Patterns of equilibrium abundance (a, b) for the wild pollinator (blue lines), plant (green solid lines), the managed honey bee (black dashed lines), and pollination rewards, *X_t_* (green dotted lines). Panels c and d show the two equilibrium plant traits associated with each pollinator trait (matched by line type). Here, we assume there is no direct competition between pollinators (*c*_1_ = *c*_2_ = 0). Panels a and c represent final equilibrium abundances (a) and traits (c) for a range of the managed honey bee carrying capacity (*k*_2_) values for the wild pollinator. Panels b and d represent final equilibrium abundances (b) and traits (d) for a range of mutualism benefits received by the honey bee (*θ*_2_). Other parameter values are presented in Table 1

In the absence of direct competition between pollinators (*c*_1_ = *c*_2_ = 0), there exists only indirect scramble competition for resources. In this scenario, increasing the managed honey bee's carrying capacity (*k*_2_) or plant benefits received (*θ*_2_) reduced wild pollinator abundance and increased the honey bee and plant abundance (Figure 3a,b). In Figure 3, the overall trends in abundance are similar, but weaker than scenarios where pollinators compete directly (Figure 2). Indeed, pollination rewards decreased rather than increased in response to higher honey bee carrying capacity or plant benefit received, due to greater consumption of the plant benefits (green dotted lines, Figure 3a,b). Furthermore, the wild pollinator and managed honey bee coexisted across the full range of parameter variation, presumably due to weaker negative interactions between the two pollinators.

### 3.2 Effects of evolutionary costs on coexistence

**FIGURE 4:**
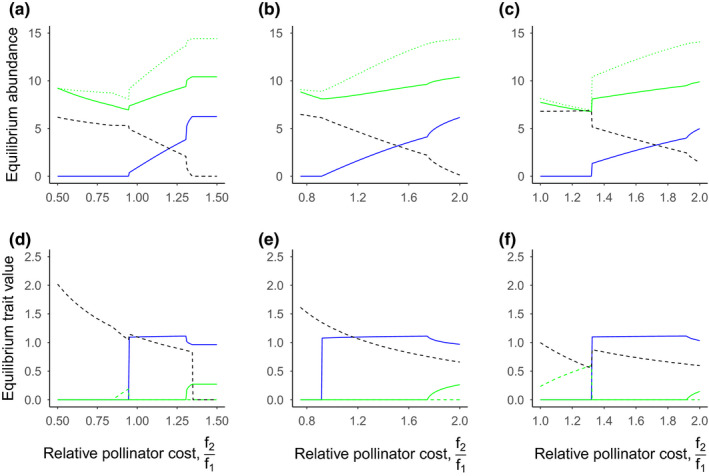
Effects of managed honey bee costs on pollinator and plant abundance and traits. Patterns of equilibrium abundance (a, b, c), and equilibrium trait values (d, e, f) for the wild pollinator (blue lines), plant (green solid lines), the managed honey bee (black dashed lines), and pollination rewards (panels a, b and c only), *X_t_* (green dotted lines). Panels d, e, and f show the two plant traits associated with each pollinator (matched by line type). The wild pollinator is affected by per capita competition more than the honey bee (*c*_1_ = 0.95, *c*_2_ = 0.75) (a,d), has a lower carrying capacity than the honey bee (*k*_1_ = 1.5, *k*_2_ = 1.75) (b,e), or benefits less from each plant visit (*θ*_1_ = 0.005, *θ*_2_ = 0.075). Parameter values varied along the *x*‐axis are the trait cost for the honey bee (*f*_2_) relative to the wild pollinator (*f*_1_) in their investment in the plant. Other parameter values are presented in Table 1

**FIGURE 5:**
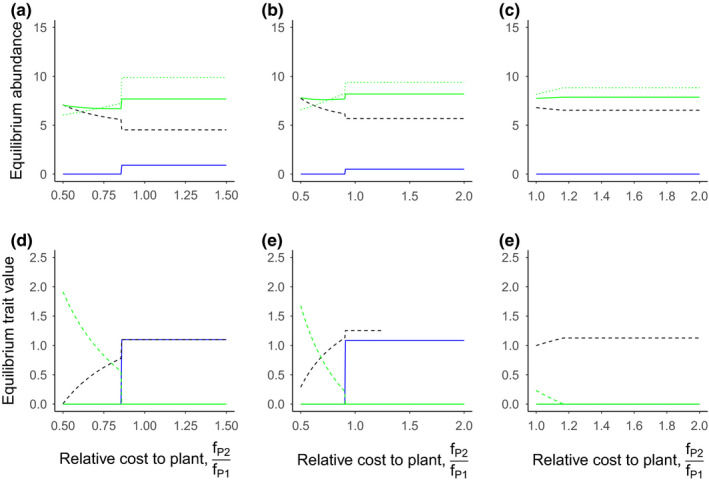
Effects of plant costs on pollinator and plant abundance and traits. Patterns of equilibrium abundance (a, b, c), and equilibrium trait values (d, e, f) for the wild pollinator (blue lines), plant (green solid lines), the managed honey bee (black dashed lines), and pollination rewards (panels a, b, and c only), *X_t_* (green dotted lines). Panels d, e, and f show the two plant traits associated with each pollinator (matched by line type). The wild pollinator is affected by per capita competition more than the honey bee (*c*_1_ = 0.95, *c*_2_ = 0.75) (a,d), has a lower carrying capacity than the honey bee (*k*_1_ = 1.5, *k*_2_ = 1.75) (b,e), or benefits less from each plant visit (*θ*_1_ = 0.005, *θ*_2_ = 0.075) (c,f). Parameter values varied along the *x*‐axis are the trait cost for the plant investing in the honey bee (*f_P_*_2_) relative to the wild pollinator (*f_P_*_1_). Other parameter values are presented in Table 1

We also evaluated the impacts of evolutionary costs to the pollinator (Figure 4) and plant (Figure 5) on pollinator coexistence. Specifically, we explore the effects of these costs in three scenarios:

1. The wild pollinator is affected by per capita competition more than the managed honey bee (*c*_1_ = 1, *c*_2_ = 0.75),
2. The managed honey bee has a higher carrying capacity than the wild pollinator (*k*_1_ = 1.5, *k*_2_ = 1.75), and
3. The wild pollinator benefits less from each plant visit (*θ*_1_ = 0.005, *θ*_2_ = 0.075).

In each scenario, coexistence was promoted by higher mutualism investment costs to the managed honey bee (*f*_2_) (Figure 4) by reducing the honey bee's abundance and trait values, allowing the wild pollinator to persist. When *f*_2_ was sufficiently greater than *f*_1_, the managed honey bee was displaced. In scenario 1 (strong per capita interspecific competition on wild pollinators) the coexistence space was small (Figure 4a), compared to scenarios 2 and 3 (Figure 4b,c), with the wild pollinator and managed honey bee displaced at the lower and upper ends of the range, respectively (Figure 4a). This is likely because, in contrast to the direct effects of managed honey bees, the wild pollinator indirectly benefits from higher honey bee abundance by increasing the abundance of its plant mutualist. Particularly, high costs to the plant of investing in the managed honey bee (*f*_P2_) can also rescue the wild pollinator from displacement driven by direct competition (high *c*_1_; Figure 5a) or high honey bee carrying capacity (high *k*_2_; Figure 5b). This coexistence is driven by reduced selection pressure for the plant to switch from plant traits associated with the wild pollinator to those associated with the managed honey bee. While the wild pollinator is rescued in these scenarios, the managed honey bee remains the dominant mutualist with much higher abundance, regardless of the cost to the plant and investment in traits (Figure 5). However, these costs are not enough to allow coexistence when managed honey bees benefit more from each plant visit than wild pollinators (*θ*_2_; Figure 5c).

## 4 DISCUSSION

Field studies often suggest that managed pollinators such as European honey bees can reduce wild pollinator diversity and plant reproduction (Geldmann & Gonzalez‐Varo, 2018; Mallinger et al., 2017). Here, we provide an evolutionary perspective to interpret these findings. Our results suggest that evolutionary changes in plants, and pollinators can mediate and even exacerbate the risks of wild pollinator displacement associated with managed pollinators. In one scenario, evolution of the plant to be more attractive to honey bees can magnify the negative effects of honey bees on wild pollinators, promoting displacement of the wild pollinator. In contrast, wild pollinator persistence is more likely when evolutionary costs associated with locating plants, or attracting honey bees, are relatively high for the honey bees or plants, respectively.

Our model provides a framework to assess how honey bees may cause mutualism breakdown between wild pollinators and plants. A key remaining question is what role phenotypic plasticity and/or evolutionary changes in plants will have on interacting populations of wild pollinators and managed honey bees and plants they pollinate. In areas where honey bees have been introduced, maintained by humans, or persist in feral colonies over long periods, evaluations of traits and visitation rates by each species on timescales over which pollinators and plants can coevolve may lead to novel insights. Evaluations in Tibet suggest that lotus plants close to apiaries have developed traits which increase honey bee attraction while reducing the production of nectar rewards, but the impacts of these new traits to attract honey bees on wild pollinator populations are unknown (Mu et al., 2014). Where long‐term observations and experiments are not possible, combining molecular and field approaches may lead to novel insights into the potential for plant phenotypic change described in our models. For example, plant visitation rates may be evaluated by observation studies, identifying pollen collected off pollinators, and/or from traces of pollinator DNA left behind in floral nectar (Vamosi, Gong, Adamowicz, & Packer, 2017). Associating interaction rates with particular plant traits or genotypes could be a powerful approach to identifying the phenotypic or genetic variance associated with plants recruiting each type of pollinator. Selection pressure could be quantified by measuring plant reproduction and relating it to changes in pollinator density. Taken together, these approaches could evaluate potential for phenotypic changes in plant populations and provide insights into the long‐term effects managed pollinators such as honey bees will have on wild pollinators and plants.

While pollinator attraction is critical for plants, particularly in self‐incompatible species, plants evolve in response to a range of selection pressures such as variation in water availability (Kooyers, 2015), temperature (Hedhly, Hormaza, & Herrero, 2009), and herbivory (Strauss & Agrawal, 1999). Plants do not respond to these selection pressures in isolation, and evolutionary responses to one selection pressure can reduce the plants ability to respond to another (Hedhly et al., 2009; Ramos & Schiestl, 2019). Thus, alternative selection pressures may alter the fitness costs associated with pollinator attraction. This is supported by recent research suggesting that plant traits that attract pollinators can also attract herbivores (Ramos & Schiestl, 2019). For example, brassica plants evolving in the presence of bumblebees exhibited larger flowers with greater flower volatility that improved attractiveness to pollinators compared to plants evolving in isolation (Ramos & Schiestl, 2019). Evolution of more attractive flowers, however, was diminished when herbivores were present due to selection pressure the herbivores imposed on plant traits (Ramos & Schiestl, 2019). However, plant traits associated with bumble bee attraction were not promoted by evolution in the presence of hoverfly pollinators (Gervasi & Schiestl, 2017). Thus, the evolution of plants traits that attract one pollinator over another may be a complex process, where costs associated with attracting each pollinator are governed by both physiological or demographic limitations and other selection pressures. Nonetheless, our results suggest that a better understanding of how these traits correspond with honey bee attraction in particular may improve our understanding of the potential for plant evolution to exacerbate the effects of honey bees on wild pollinator populations and the plants the wild pollinators pollinate.

Here, we evaluated a simple model where we assumed that plants have two traits, each associated with a different pollinator. Although we assumed that investing in both plant traits simultaneously was particularly expensive, for simplicity, we did not consider genetic correlations between the plant traits (Ashman & Majetic, 2006; Conner, 2002). The effects of such genetic correlations would likely depend on the mathematical form that such correlations take. However, in general positive correlations would likely reduce the potential for plant evolution to exacerbate pollinator declines, because it would inhibit plant switches from traits associated with wild pollinators to traits associated with managed honey bees. Conner et al. (2011), though found that even flower traits with strong genetic correlation can exhibit independent evolution when selected upon. The authors used artificial selection to select for lengths and filaments and corolla tubes in wild radishes (*Raphanus raphanistrum*) and observed independent evolution of each trait, despite strong pleiotropic genetic correlation of the two traits. Therefore, the effects of genetic correlations (not included in our model) on evolutionary plant responses to pollinators are needed to fully understand the impacts of plant evolution and pollinator introduction on wild pollinator communities.

Pollinator and/or plant adaptation to changing species abundances and traits can also lead to network rewiring, inducing changes in pollination networks (CaraDonna et al., 2017). Our model provides a foundation to build more complex network models to evaluate managed pollinator species’ direct and indirect effects on wild pollinator–plant mutualisms (Burkle et al., 2013; CaraDonna et al., 2017; Guimaraes et al., 2017; Tylianakis, 2008). While we only consider evolution of plant traits, we do not assume any particular genetic structure coding the traits, and only assume that plants and pollinator population traits respond to selection pressure. Therefore, we expect plastic trait change to behave similarly to evolutionary change in this manner, and our model may also provide insight into network rewiring based on phenotypic plasticity. Pre‐existing plant–pollinator networks could be used to inform and build on our model network structure and develop hypotheses for the types of network rewiring induced by honey bee introduction (Bartomeus, 2013; CaraDonna et al., 2017). Furthermore, while we model only one (pollinator), or two (plant) traits that alter pollinator visitation rates, these traits are likely complex and mediated by a diverse suite of genes. Through the development of simple eco‐evolutionary models such as the one described here, we can parsimoniously improve our understanding of how pollinator attraction and foraging behavior impact pollinator–plant coevolution.

## 5 CONCLUSION

Our analyses were inspired by ecological studies documenting short‐term effects of honey bees on wild pollinators (Geldmann & Gonzalez‐Varo, 2018; Geslin et al., 2017; Mallinger et al., 2017), but whether these effects persist over evolutionary time scales has proven challenging to determine. Our model framework presents a novel opportunity to develop hypotheses for how plant evolution alters the persistence of these effects. Specifically, plant evolution is likely to exaggerate negative impacts on wild pollinators as plants switch to attract more honey bees. Persistence of the wild pollinator is promoted when plant evolution is limited by high costs associated with plant traits involved in attracting honey bees or honey bee traits that increase plant visitation. More broadly, a better understanding of these costs and plants’ ability to adapt to pollinator introductions will allow us to better predict the long‐term implications of managed honey bees on wild pollinators.

## CONFLICT OF INTERESTS

We have no competing interests.

## AUTHOR CONTRIBUTIONS

JM, EB, and TN conceived the study. JM and TD conducted the analysis with feedback from EB and DC. JM wrote the manuscript with substantial edits from all authors.

## ETHICAL STATEMENT

No research was conducted on plants or animals as part of this research.

## Supporting information

Appendix S1

## Data Availability

Representative code for running our model is presented in Dryad https://doi.org/10.5061/dryad.bcc2fqz8d.
