# Supplementary material for: Plant evolution can mediate negative effects from honey bees on wild pollinators
Source: Ecol Evol. 2020 Apr 12;10(10):4407–18. doi: 10.1002/ece3.6207 (PMC7246215; doi:10.1002/ece3.6207)
Supplement: Supplementary file 1 — Appendix S1 [file ECE3-10-4407-s001.docx]

APPENDIX

Derivation of equations for plant trait i:

$$u_{i,t+1}=u_{i,t}+\frac{1}{W_{P}}\frac{\delta W_{P}}{\delta u_{1}}{\sigma_{P1}}^{2}$$

$$=u_{i,t}+\left[ \frac{e^{r_{P}-\frac{r_{P}}{k_{P}-f_{Pi}u_{i,t}-f_{Pj}u_{j,t}}P_{t}+\vartheta_{Pi}N_{i,t}ln\left( v_{i,t}+{d_{i}u}_{i,t} \right)+\vartheta_{Pj}N_{j,t}ln\left( v_{j,t}+{d_{j}u}_{j,t} \right)}}{e^{r_{P}-\frac{r_{P}}{k_{P}-f_{Pi}u_{i,t}-f_{Pj}u_{j,t}}P_{t}+\vartheta_{Pi}N_{i,t}ln\left( v_{i,t}+{d_{i}u}_{i,t} \right)+\vartheta_{Pj}N_{j,t}ln\left( v_{j,t}+{d_{j}u}_{j,t} \right)}}\left( \left( -\frac{r_{P}P_{t}f_{P1}}{\left( k_{P}-f_{Pi}u_{i,t}-f_{Pj}u_{j,t} \right)^{2}} \right)+\frac{\vartheta_{Pi}N_{1,t}d_{i}}{{(v}_{i,t}+{du}_{i,t}+1)\left( a_{01}+P_{t} \right)} \right) \right]{\sigma_{P1}}^{2}$$

$$=u_{i,t}+\left[ \left( -\frac{r_{P}P_{t}f_{P1}}{\left( k_{P}-f_{Pi}u_{i,t}-f_{Pj}u_{j,t} \right)^{2}} \right)+\frac{\vartheta_{P1}N_{1,t}d_{i}}{{(v}_{i,t}+{du}_{i,t}+1)\left( a_{01}+P_{t} \right)} \right]{\sigma_{P1}}^{2}$$

Substitute in *b*_i_:

$$=u_{1,t}+\left[ \left( -\frac{r_{P}P_{t}f_{P1}}{{b_{P}\left( u_{1,t}, u_{2,t} \right)}^{2}} \right)+\frac{\vartheta_{P1}N_{1,t}d_{i}}{{(v}_{i,t}+{du}_{i,t}+1)\left( a_{01}+P_{t} \right)} \right]{\sigma_{P1}}^{2}$$

Equation for trait change for pollinator species *i*:

$$v_{i,t+1}=v_{i,t}+\frac{1}{W_{Ni}}\frac{\delta W_{Ni}}{\delta v_{i}}{\sigma_{i}}^{2}$$

$$=v_{i,t}+\left[ \frac{e^{r_{i}-\frac{r_{i}}{k_{i}-f_{i}v_{i}}\left( N_{i,t}+c_{i}N_{j,t} \right)+(\vartheta_{i}X_{t}\frac{ln\left( v_{i,t}+{du}_{i,t}+1 \right)}{a_{0i}+P_{t}})}}{e^{r_{i}-\frac{r_{i}}{k_{i}-f_{i}v_{i}}\left( N_{i,t}+c_{i}N_{j,t} \right)+(\vartheta_{i}X_{t}\frac{ln\left( v_{i,t}+{du}_{i,t}+1 \right)}{a_{0i}+P_{t}})}}\times\left( \frac{-r_{i}{(N}_{i,t}+c_{i}N_{j,t})f_{i}}{\left( k_{i}-f_{i}v_{i} \right)^{2}}+\frac{\vartheta_{i}X_{t}}{\left( v_{i,t}+{du}_{i,t}+1 \right)\left( a_{0i}+P_{t} \right)} \right) \right]{\sigma_{i}}^{2}$$

$$=v_{i,t}+\left[ \frac{-r_{i}{(N}_{i,t}+c_{i}N_{j,t})f_{i}}{\left( k_{i}-f_{i}v_{i} \right)^{2}}+\frac{\vartheta_{i}X_{t}}{\left( v_{i,t}+{du}_{i,t}+1 \right)\left( a_{0i}+P_{t} \right)} \right]{\sigma_{i}}^{2}$$

Substitute in *b*_i_:

$$= v_{i,t}+\left[ \left( -\frac{r_{i}{(N}_{i,t}+c_{i}N_{j,t})f_{i}}{{b_{i}\left( v_{i,t} \right)}^{2}} \right)+\frac{\vartheta_{i}X_{t}}{\left( v_{i,t}+d_{i}u_{i,t}+1 \right)\left( a_{0i}+P_{t} \right)} \right]{\sigma_{i}}^{2}$$
